# Oocyte Cryopreservation in Women with Ovarian Endometriosis

**DOI:** 10.3390/jcm12216767

**Published:** 2023-10-26

**Authors:** Judith-Marie Mifsud, Livia Pellegrini, Mauro Cozzolino

**Affiliations:** 1 IVIRMA Global Research Alliance, IVIRMA Roma, 00169 Rome, Italy; judithmmifsud@gmail.com (J.-M.M.); livia.pellegrini@ivirma.com (L.P.); 2 IVIRMA Global Research Alliance, IVI Foundation, Instituto de Investigación Sanitaria La Fe (IIS La Fe), 46026 Valencia, Spain

**Keywords:** endometrioma, endometriosis, endometriosis surgery, ovarian cystectomy, fertility preservation, oocyte vitrification, egg freezing

## Abstract

Ovarian endometriosis is a gynecological condition that is closely associated with infertility—from its pathogenesis to treatment modalities, this condition presents a challenge both for patients and clinicians alike when seeking conception, due to low AMH levels, peritoneal inflammation, and the inadvertent removal of healthy ovarian parenchyma at surgery. In fact, around half of endometriosis patients seeking fertility require tertiary-level assisted reproduction techniques to achieve a live birth. Oocyte cryopreservation, a procedure initially designed for oncology patients, has emerged over recent years as a very promising treatment strategy for patients who have been diagnosed with ovarian endometriosis in order to preserve their fertility and obtain a live birth at a later stage in their lives. Counseling patients about oocyte preservation techniques at an early stage in the diagnosis, ideally before the age of 35 and especially prior to any surgical treatment, provides an excellent opportunity to discuss future fertility and the benefits associated with oocyte cryopreservation.

## 1. Introduction

Endometriosis is a chronic inflammatory gynecological condition and is found in about 30–50% of infertile women [1]. The pathogenesis of endometriosis is complex and multifactorial [2] and is responsible for a detrimental effect on ovulation and oocyte quality, tubal function, fertilization, and implantation, ultimately leading to infertility [3]. Around 50% of endometriosis patients may ultimately require in vitro fertilization (IVF) procedures to achieve a live birth [4]. There are three main types of abdominal/pelvic endometriosis, namely superficial peritoneal endometriosis (SPE), ovarian endometrioma (OMA), and deep infiltrating endometriosis (DIE) [5,6].

SPE is found in 15–50% of women diagnosed with endometriosis [7,8]. There are three lesions associated with SPE which may be identified laparoscopically as red, black, or white, colors which represent the progression of the disease from vascularized active lesions to advanced disease, to its quiescent phase [5]. Such presence of ectopic lesions on the peritoneum reduces the chances of spontaneous fertilization threefold [9].

OMAs are present in up to 44% of women with endometriosis, making it the most common subtype of endometriosis [10]. They are found in 50% of women treated with infertility [11]. Their presence significantly lowers serum anti-Müllerian hormone (AMH) levels [12,13], with significant reductions reported among the presence of larger OMAs and bilateral lesions [14]. Moreover, women with OMAs experience a faster decline in serum AMH levels compared to their age-matched counterparts [15,16]. These features are attributable to several complex interacting factors, including increased primordial follicle activation, that give OMAs this inherent characteristic of increased ovarian ageing [17].

DIE refers to abdominal/pelvic lesions more than 5 mm below the serosal/peritoneal surface [18], and these are present in about 20% of women affected by endometriosis [19] DIE commonly develops in the uterosacral ligament, urinary tract, rectovaginal, and retrocervical areas and may manifest as infertility [20]. DIE also has a significant impairment of sexual activity especially in the presence of partial or total infiltration of the rectovaginal septum [21].

Although it is considered a benign disease, malignant transformation may occur, and endometriosis is associated with ovarian, thyroid, breast, ovarian, and even vaginal malignancy [22,23]. 

Surgical removal of OMA is one of the commonly described treatment modalities for this disease. Unfortunately, this has been associated with an irreversible negative impact on ovarian reserve, as evidenced by a significant postoperative fall in circulating AMH [24,25], especially in cases of bilateral OMA surgery [13]. Surgical techniques undoubtedly lead to the inadvertent removal of healthy ovarian parenchyma, which is proportional to the OMA size [26]. This effect seems to be inevitable, even in the hands of experienced laparoscopic surgeons [27].

Oocyte vitrification has been recognized as a distinguished method of fertility preservation (FP) worldwide that serves to preserve female gametes for potential future motherhood in cases where reproductive function is threatened for various reasons [28]. Since the birth of the first documented human pregnancy from oocyte cryopreservation (OOC) in 1986 [29], there has been a significant improvement in vitrification techniques—including ultrarapid vitrification protocols—which has led to a crucial improvement in oocyte survival and clinical pregnancy rates [30,31]. A recent systematic review also confirmed that oocyte vitrification techniques do not present an increased risk of adverse neonatal outcomes [32]. Younger patients undergoing OOC seem to have higher oocyte yields, lower ovarian stimulation cycles, and higher live birth rates compared to those performed in an older age group [33], which needs to be considered when patients, especially those suffering from fertility-impairing conditions, present to the clinic. The difficulty encountered in most studies is the low percentage of women who return to use their cryopreserved oocytes, which currently stands at 7.4% [34]. Apart from the benefit of fertility preservation in this specific population, studies highlighted a potential benefit of freeze-all strategy in endometriosis patients [35]. 

In view of the negative correlation of OMAs, including their surgical management, on fertility, it has been postulated that fertility preservation (FP) is a valid treatment to be considered in the holistic management of endometriosis, with individualization of the treatment plan depending on the patient’s age [36]. The overall true benefit of fertility preservation in women with endometriosis remains relatively unknown, with the current European Society for Human Reproduction and Endocrinology (ESHRE) guideline recently recommending that women with extensive ovarian endometriosis undergo detailed discussions regarding the pros and cons of fertility preservation with their clinician [37].

The updated European Society for Human Reproduction and Endocrinology (ESHRE) guideline on endometriosis and its management claims that the cost-effectiveness of fertility preservation and whether this procedure should be offered to all women with endometriosis remains unclear [37]. ESHRE recommends that clinicians should discuss the pros and cons of fertility preservation in cases of women with ‘extensive ovarian endometriosis’ (strong recommendation) and that studies focus on the identification of women who have greater chances of requiring assisted reproductive technology (ART) treatments in the future—either due to the diagnosis itself or following the need for surgery—to help support the use of fertility preservation in selected women with endometriosis. In this review, we will challenge the reasons why OS is an important management option for women with endometriosis.

## 2. Materials and Methods

**Figure 1:**
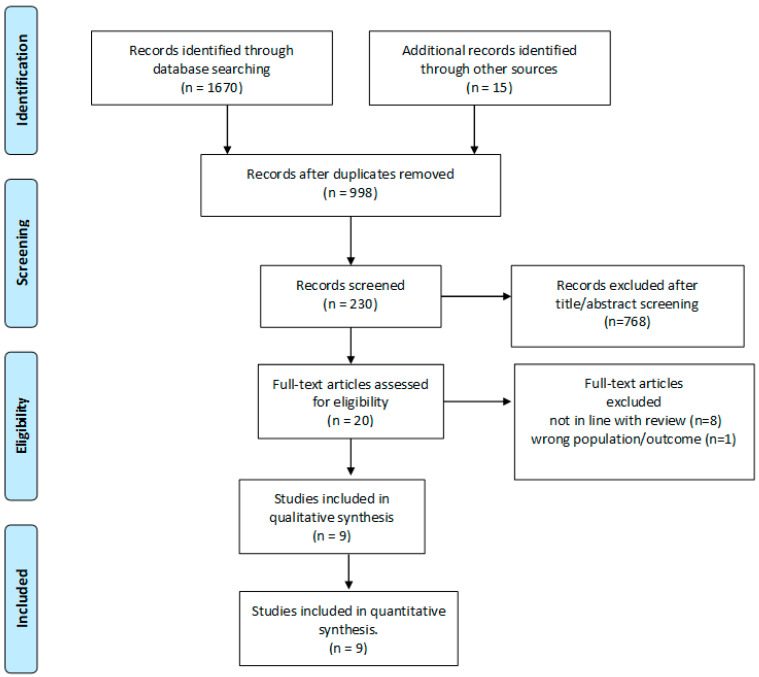
PRISMA flow diagram.

A search in PUBMED and MEDLINE for peer-reviewed papers published in English from 2000 through December 2022 was conducted, and articles related to oocyte freezing for fertility preservation in women with endometriosis were included. The following search words were used: “endometriosis”, “endometrioma”, “endometriotic”, “oocyte retrieval”, “fertility preservation”, “oocyte preservation”, “oocyte cryopreservation”, “fertility preservation”, “oocyte freezing”, “oocyte vitrification”, and “oocyte retrieval”. References from selected papers and dedicated reviews were assessed for any missed studies. Abstracts, conference proceedings, review articles, and case-reports were excluded, as were studies that included pediatric, adolescent, or transgender patients. In Figure 1 is reported the flow-chart of the study included in the review.

## 3. Ovarian Reserve in Endometriosis

An important tool in fertility medicine is the estimation of serum anti-Müllerian hormone (AMH) levels, a hormone which is synthesized by granulosa cells surrounding ovarian follicles [38]. Apart from being an ovarian reserve marker, it allows the physician to individualize gonadotrophin dosing in assisted reproduction techniques [39]. It is well-known that endometriosis is associated with low AMH [40]. A recent systematic review has concluded that the presence of endometriosis significantly decreases AMH levels when compared to controls [41]. In addition, subgroup analysis assessed the antral follicle count (AFC) of ovaries containing endometriomas and found that AFC in the affected ovary was significantly lower than that of the contralateral ovary before surgery, supporting the hypothesis that most of the damage to the ovarian reserve existed before surgery [41].

AMH is reduced in patients with OMA compared to patients with other benign ovarian cysts or with healthy ovaries [12]. The impact of endometriosis on the ovarian reserve is of particular concern since endometriotic cysts contain potentially toxic substances—such as free iron—that may diffuse through the cyst wall and damage the ovarian reserve [42]. Moreover, there is a potential detrimental mechanical effect on the exposed ovarian cortex, following the long-lasting stretching effect consequent to the presence of these cysts [42]. In addition, a significant reduction of primordial follicles in the ovarian cortex of women affected by the disease has also been histologically confirmed [43,44].

The presence of OMA has been associated with accelerated ovarian ageing with the consequence of earlier menopause due to premature ovarian insufficiency (POI). This effect is possibly due to the hyperactivation of primordial follicles in the presence of nearby endometriomas, leading to ovarian exhaustion [45].

Among infertility patients with endometriosis, with and without a history of ovarian surgery, ovarian reserve markers were worse (lower AMH and higher FSH) [40]. In addition, impairment of granulosa cell function in endometriosis is reflected by a decreased inhibin B secretion in patients with endometriosis. Inhibin B may serve as an alternate marker to assess follicular development or to predict the number of oocytes retrieved [46]. Recent studies on infertile women with endometriosis have isolated the presence of the variant T allele in the follicle-stimulating hormone (FSH) receptor β-subunit (*FSHB)*:c.−211G>T single nucleotide variants (SNV) affected luteinizing hormone (LH) levels in women with overall endometriosis and minimal/mild disease. *FSHR*:c.919G>A SNV affects FSH levels in women with overall endometriosis and the number of oocytes retrieved in those with moderate/severe endometriosis, while the *FSHR*:c.2039G>A SNV affected FSH levels in women with overall endometriosis [47]. Table 1 shows a list of studies that have examined the outcomes of fertility preservation in endometriosis patients.

## 4. Effects of Surgery for Ovarian Endometriosis on Ovarian Reserve

It is well known that ovarian OMA surgery negatively impacts AMH levels with several studies supporting this view [52,56,57]. Although the mechanisms of surgical damage on the ovarian reserve are not fully elucidated, several processes have been suggested, including the inadvertent removal of primordial follicles adjacent to the endometrioma, excessive use of electrocoagulation causing damage to ‘healthy’ ovarian tissue, the interruption of ovarian vascularization, the local inflammatory reaction, and the burn-out effect [58].

Santulli [52] compared AMH in patients with OMA who had undergone previous endometriosis surgery and found that the median AMH concentration was significantly higher (2.1 ± 1.6 ng/ml versus 1.6 ± 1.9 ng/mL; *p* < 0.001) together with a higher AFC (13.2 ± 8.2 versus 10.3 ± 5.7; *p* = 0.028) in patients who had no previous surgery compared to the women in the ‘previous surgery’ group.

This finding was reproduced in the study by Elizur [55] where women in the ‘no previous surgery’ groups had a higher AMH level (0.69 ng/mL versus 2.1 ng/mL, *p* < 0.001) than those who had undergone prior OMA surgery. The median AMH concentration was lower (0.86 ng/mL versus 2.6 ng/mL, *p* < 0.001) among patients who had prior surgery and had OMA larger than 4 cm, compared with those who did not.

Cobo [36] also concluded that AMH levels were lower in the surgical patients, with the lowest AMH levels observed in those who underwent bilateral surgery (NS), even though they were the youngest cohort included (33.4 versus 36.7 years).

## 5. Surgical Techniques That May Help to Reduce Ovarian Damage in Endometrioma Surgery

An interesting recent systematic review and meta-analysis on the effect of laparoscopic endometrioma surgery on AMH levels by Moreno-Sepulveda [57] discussed that there is a significant decrease in short-, medium- and long-term post-operative AMH levels when compared to baseline AMH. No differences were observed between short- and long-term post-operative AMH levels. Interestingly, the authors suggested a non-significant recovery of AMH levels after one year of follow-up. A significant decrease in post-operative AMH was observed in the short-, medium- and long-term periods with bilateral endometrioma surgery when compared to unilateral endometrioma surgery. In turn, the decrease in AMH was significantly greater when the size of the endometriomas exceeded 7 cm [57].

The type of laparoscopic surgery carried out also reflected the change in AMH levels post-surgery. Post-operative AMH levels are significantly lower after bilateral cystectomy compared to vaporization using bipolar energy. Lower post-operative AMH levels were also observed after laser vaporization when compared to cystectomy procedures, while no difference was observed when comparing post-operative AMH levels of patients submitted to unilateral cystectomy versus bipolar energy vaporization. Post-operative AMH also decreased significantly with the use of hemostasis using bipolar energy, compared to suturing with the use of hemostatic agents. On the other hand, this difference was not significant when compared to hemostasis with ultrasound [57].

Endometriosis is a chronic disorder with a significant tendency to recur, and rates of recurrence include 22% of women at 2 years and 40–50% at 5 years (about 10% rate per year) [59]. All types of endometriotic lesions have a high rate of recurrence after conservative surgery. It has been proposed that recurrence rates of endometriosis after surgery occur due to cyst fluid leakage of ovarian endometriotic cyst fluid, which contains living endometrial cells with high adhesion ability, which may contribute to the recurrence of endometriosis after surgical excision [60]. This poses a significant further strain on the management of endometriosis.

A meta-analysis showed that bipolar desiccation (BP) is more detrimental to ovarian reserve when compared to alternative hemostatic methods, with evidence favoring hemostatic sealant (HS) (high-quality evidence) and low-quality evidence favoring sutures over BD. Compared with BD, alternative hemostatic methods are associated with significantly less decline in ovarian reserve [61]. 

The use of the FloSeal hemostatic agent when performing ovarian cystectomy for ovarian endometrioma yielded a higher AFC at one year but no difference in FSH or AMH levels compared to hemostasis with diathermy. In addition, the spontaneous pregnancy rate and endometrioma recurrence were not significantly different between the two [62].

## 6. Ovarian Stimulation in Women with Endometriosis

Patients with endometriosis required higher total doses of gonadotrophins when compared to infertile women due to other causes [51,54]. Patients with unilateral OMAs required higher doses of gonadotrophins compared to those with bilateral OMAs (2594 versus 2368; *p* ≤ 0.001), and ovarian stimulation did not alter the size of the OMAs [51]. In fertility preservation cycles for women with endometriosis, the duration of stimulation was similar when comparing the presence of unilateral and bilateral OMAs [51,54]. There was no difference in the number of days of stimulation when comparing superficial endometriosis, deep endometriosis, and OMAs [49].

However, it is unclear if women with endometriosis need more days of ovarian stimulation; in fact, women with OMA required a slightly longer duration of stimulation compared to infertile women due to other causes (8.0 versus 7.3 days, *p* = 0.106) [51], but in another study, both groups of women needed similar days of stimulation (8.0 versus 8.0 days; *p* = 0.841) [54].

When comparing OMAs to other benign cysts, the duration of stimulation was very similar [51,53,54].

Similar days of stimulation were required in both antagonist stimulation protocols and progestin-primed ovarian stimulation (PPOS) protocols [50]. It was observed that a similar number of retrieved oocytes and vitrified mature oocytes were obtained from either stimulation protocol. No complications were observed during either stimulation or oocyte retrieval procedures, and both protocols were equivalent in the outcomes of preservation.

The total amount of gonadotrophins used was significantly higher in patients who underwent previous endometriosis surgery compared to those who did not have previous surgery (4950 versus 3000 IU; *p* = 0.001) [38]; however, this finding was not reproduced in other studies [49,52]. There is a significant difference noted when comparing operated OMAs above 4 cm versus unoperated endometriomas larger than 4 cm (2850 versus 4950 IU, respectively; *p* ≤ 0.001) [55]. Interestingly, the total gonadotrophin dose also increased markedly with the presence of unoperated endometriomas smaller than 4 cm when compared to unoperated endometriomas larger than 4 cm (4050 versus 2850 IU; *p* = 0.003) [55]. The presence of large endometriomas (≥5 cm) at the time of IVF has been found to significantly decrease the number of oocytes retrieved compared with the contralateral healthy ovaries [63].

A recent randomized controlled trial revealed that compared with GnRHa, dienogest is more effective in preserving ovarian reserve after cystectomy of ovarian endometrioma. One year post-operatively, over 60% of the patients in the dienogest group retained over 70% of their pretreatment AMH levels, compared to zero patients within the GnRHa group. Interleukin-6 (IL-6), a key cytokine involved in inflammation, was found in lower levels in the dienogest group, hence showing that this treatment lowers the inflammatory response during the perioperative period and other endometriosis-related inflammatory reactions [64].

## 7. Outcomes of Fertility Preservation

Infertile women with OMAs had significantly fewer oocytes retrieved when compared to infertile women due to other causes [41,42]. The number of retrieved oocytes was significantly higher in OMA patients 35 years or younger when compared to those over 35 [36].

As expected, evidence of prior endometriosis surgery decreased the yield of retrieved oocytes, with statistically significant results [35,52,55]. Although within the group of surgical patients, the presence of bilateral endometriomas compared to the presence of unilateral endometriomas yielded fewer oocytes without statistical significance [51,54], Cobo [36] found no difference between these two groups; a possible explanation is that out of the 14.6% of women who underwent unilateral oophorectomy, 45.5% also had a contralateral cystectomy, with 4.6% having undergone multiple unilateral cystectomies [36]. There was no difference in the number of retrieved oocytes with the presence of OMAs smaller or larger than 4 cm in those women who had not undergone previous surgery (NS) [55].

The number of retrieved MII oocytes followed a similar trend. Multivariate logistic regression analysis of a number of MII oocytes retrieved demonstrated a 51.7% (95% CI –26.1 to –68.5, *p* = 0.001) reduction in women with OMA who had surgery before FP compared with those who did not, and all variables of ovarian reserve and responsiveness in women with OMA with previous surgery before fertility preservation showed significantly poorer results than those who did not [55]. Similarly, the mean number of oocytes retrieved after the first ovarian stimulation cycle and the total number of oocytes retrieved per woman were significantly higher in the women without a previous history of surgery compared with the women who had previously undergone surgery for ovarian endometriosis (9.5 ± 7.2 versus 5.7 ± 4.7; *p* = 0.002 and 14.7 ± 8.3 versus 10.6 ± 7.0; *p* = 0.013, respectively) [52]. The AMH serum level and the gravidity were positively correlated with an increase in the number of oocytes retrieved (coefficient 1.65; 95% CI 1.13 to 2.17; *p* < 0.001 and coefficient 3.30; 95% CI 0.91 to 5.68; *p* = 0.007, respectively) [52].

In the study by Raad [49], patients with superficial endometriosis and OMAs had fewer oocytes retrieved compared to those with deep infiltrating endometriosis, while Cobo [36] found no difference when comparing stage I-II versus stage III-IV endometriosis (7.4 ± 6.4 versus 7.1 ± 6.5; NS). Similarly, deep endometriosis (versus superficial endometriosis alone) was not associated with the number of retrieved oocytes (*p* > 0.05), but age (*p* = 0.001), prior ovarian surgery (*p* = 0.035), and AMH level (*p* = 0.001) were associated with the number of retrieved oocytes [50].

However, the oocyte maturation rate was lowest in OMA cycles (72.5%) when compared to superficial endometriosis and DIE, respectively (83.1% and 83.3%) [49]. The presence of bilateral OMAs also decreased the oocyte maturation rates compared to unilateral OMAs [51,54]. When comparing OMAs to other benign cysts, it was evident that fewer oocytes were retrieved in women with OMAs (NS) [53,54].

Hee Hong [54] recommends that it is necessary to counsel patients that even if a small number of oocytes were obtained in the first cycle, sufficient oocytes or embryos for future pregnancy attempts could be achieved with multiple repetitions of the procedure. In repeated oocyte retrieval cycles in women with endometrioma, the number of oocytes retrieved per cycle was not affected [51]. As the number of oocytes cryopreserved in the second cycle was similar to that cryopreserved from the first cycle, it was possible to cryopreserve about twice as many oocytes in total.

Closely related and associated with endometriosis, adenomyosis also needs to be considered since its presence negatively impacts the chances of live birth with ART treatment in endometriosis-associated infertility [65]. This may be explained by the local intraendometrial estrogen biosynthesis leading to progesterone resistance which negatively impacts implantation in adenomyosis and endometriosis [66].

## 8. Return Rate

Only the study by Cobo [36] has officially reported the return and pregnancy rate after thawing in women with endometriosis after seeking fertility preservation. A high return rate of 46.5% was observed, where 485 patients out of 1044 who had their oocytes vitrified returned to use them to attempt a pregnancy. This finding suggests that the vitrification of oocytes in this cohort of women was performed as an adjunct to the treatment of endometriosis-related infertility, rather than as a procedure done purely in order to preserve their fertility. Following these results, it can be speculated that women with endometriosis planned a pregnancy earlier than anticipated following the information given at pre-operative counseling.

In a smaller study, Santulli [52] reported a 1% return rate, with only two patients who returned to use their oocytes with both having a live birth after oocyte thawing Nonetheless, the low return rate observed in this study is probably related to the fact that women used this treatment to potentially negate the effect of the disease on future fertility, without an immediate desire for pregnancy. It is also to be noted that the mean age of the population was over 4 years younger than that in the study by Cobo [36].

## 9. Pregnancy Rates and Embryo Quality

The largest studies that gave valuable information on pregnancy and live birth rates following the thawing of vitrified oocytes in endometriosis patients are those by Cobo [28,36]. The mean age at warming was 37.3 years, and the overall oocyte survival rate was 83.2%, with the number of mean warmed oocytes reaching 8.6. There were 8.6 ± 5.6 mean warmed embryos per ICSI with an embryo survival rate of 93.6% after thawing.

In general, there were higher oocyte survival rates, implantation rates, pregnancy rates, and cumulative live birth rates (CLBR) in young (≤35 years) elective FP patients compared with endometriosis age-matched patients (*p* ≤ 0.05) [24]. Although worse results were expected in the deeper, infiltrating stages of the disease, there were no statistically significant differences in embryo quality, oocyte survival rate, pregnancy rate, or CLBR between stages I–II and III–IV [36].

In endometriosis patients up to the age of 35 years, survival rate, implantation rate, clinical pregnancy rate (CPR), and CLBR are significantly lower than age-matched women undergoing elective fertility preservation—an effect which was lost in women above the age of 35 years.

As expected, the CLBR increased as the number of vitrified oocytes used increased. Age plays an important role in this context as the percentage CLBR per oocyte retrieved is always lower in endometriosis women >35 years compared to those ≤35 years. As an example, the percentage gain nearly doubles with 5–8 oocytes retrieved from patients ≤35 years, 5.5% gain in CLBR, compared to 2.7% gain in those >35 [28].

There is no difference in CLBR when comparing endometriosis patients with women undergoing elective FP in age-matched groups, but CLBR was significantly higher in both endometriosis patients and elective FP patients when performed ≤ 35 years than when performed after the age of 35 (*p* = 0.0001) [28].

When comparing women aged ≤35 versus those >35 years, with and without surgery, previous surgery had no effect on the oocyte survival rate (NS). The clinical pregnancy rate was comparable (NS), and the ongoing pregnancy rate was higher for patients who had not had surgery (*p* < 0.05). The CLBR was statistically significantly higher in the nonsurgical group (72.5%) compared with the group of patients who underwent surgery (52.8%) [36].

Although there appears to be a decrease in the number of oocytes obtained in women with endometriosis, there seems to be no alteration in oocyte quality [67]. Analysis of embryo quality shows that in women younger than 35 years, there was a greater number of Grade A and B embryos, whilst there was a higher percentage of lower grade embryos (C, D and E) in patients above the age of 35 years. When comparing the stage of endometriosis, irrespective of age, there was a similar outcome in embryo score between both groups of endometriosis: stage I-II and III-IV [36]. These findings are like previous studies [68,69] suggesting that endometriomas and their surgical treatment compromise oocyte maturity but not embryo quality; in addition, Dongye [68] also added that even though surgery does not significantly influence the live birth rate, it seems to contribute to improve blastocyst development [68].

The Cobo study [36] was limited by the fact that most patients within the cohort were diagnosed with stages III–IV of the disease (474 versus 11), and the women in the surgically treated group were significantly younger. Hence, extrapolations on these factors to the whole population of women with endometriosis is debatable.

In the same study, a total of 218 patients failed to become pregnant after using all their vitrified oocytes, of whom 58 patients (26.6%) of mean age 36.4 years returned to attempt another pregnancy using fresh oocytes. A total of 24 infants were born, giving a CLBR per patient of 41.4%. Another 128 patients were treated with ovum donation, and 32 did not return [36].

## 10. Number of Oocytes to Cryopreserve

The procedure of oocyte cryopreservation provides no guarantee of a future pregnancy; its success is ultimately linked to the number of mature oocytes vitrified. It has been suggested that when considering the probability of live birth, the number of oocytes to cryopreserve depends on patients’ age—women under 38 years should aim to cryopreserve 15–20 oocytes, and women 38–40 years should aim to cryopreserve 25–30 oocytes [69], numbers which may be too large to accomplish considering merely patient age. In another study, in women having frozen their gametes for non-medical reasons, the vitrification of 15 mature oocytes was associated with an 85% chance of live birth in women ≤ 35 years, while for women ≥ 36 years with a total of 11 mature vitrified oocytes, CLBR plateaued at 35.6% [70]. In a more recent study, Hong [54] recommends that in general, at least 10–15 oocytes should be cryopreserved if the patient is willing to undergo FP to increase the chances of achieving a future pregnancy.

The numbers needed to treat (NNT) is a simple tool that can be used to describe statistics to patients in clinics. The NNT corresponds to 16 women with endometriosis before surgical treatment, in whom cryopreservation must be performed to guarantee one supplemental live birth [71].

## 11. Conclusions and Future Considerations

**Figure 2:**
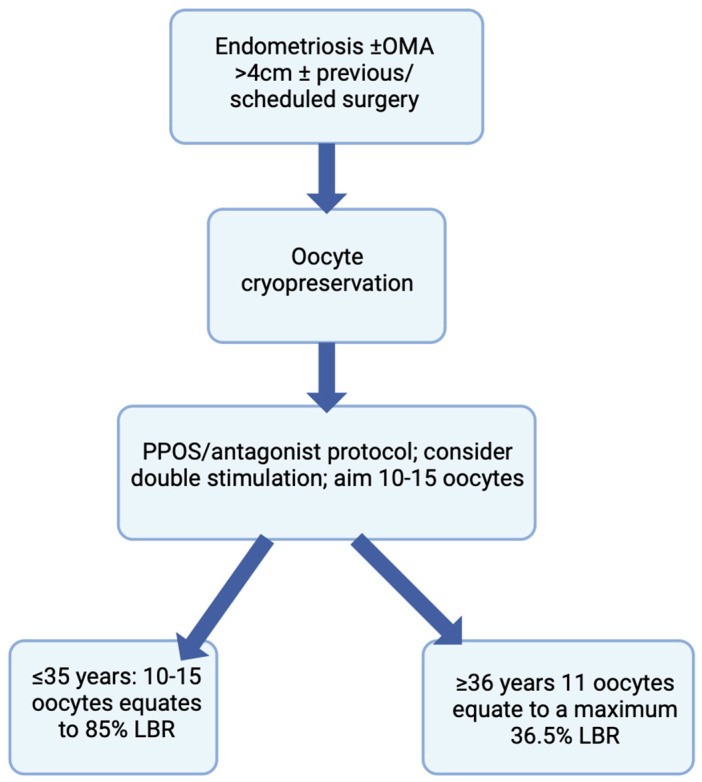
Flowchart depicting the counseling process for women with endometriosis undergoing oocyte cryopreservation.

Endometriosis is associated with a reduced ovarian reserve, with treatment modalities, such as surgery, leading to a worsening of a woman’s ovarian reserve, and possibly POI. Oocyte vitrification is a safe procedure and an excellent opportunity for these women to preserve their fertility for future use. Improved outcomes are observed when FP is accomplished before the age of 35 years and prior to any surgery involving ovarian tissue, especially in those with recurrent OMAs. Although surgery presents a quantitative decrease in oocyte yield, increasing age presents a decline in oocyte quality, and the latter has a greater effect on the success of ART outcomes, compared to other factors. Therefore, in order to gain its maximal effect, FP is most beneficial if it is undertaken before the age of 35 and prior to surgery. It is fundamental to counsel women diagnosed with endometriosis about their ovarian reserve and methods of fertility preservation, for them to make an informed choice regarding any decision they might take in the foreseeable future (Figure 2). Patients suffering from endometriosis-associated infertility should be well-informed patients, and this will facilitate the process of shared decision making, which is extremely relevant in the context of endometriosis [72]. Future considerations should include the creation of medications targeting crucial genes responsible for the aberrant pattern of expression in eutopic endometrium present in endometriosis, being an epigenetic disease, that may help treat infertility in women with this disease [73].This topic would further benefit from further randomized controlled homogenous studies using large cohorts and also including cost-effectiveness and risk-benefit ratios to further fulfill the notion that FP is an important consideration for endometriosis patients.

## Figures and Tables

**Table 1:** List of studies that examined fertility preservation in endometriosis.

First Author, Year	Study Design	Number of Endometriosis Women; Number of Cycles	Stimulation Protocol	Number of MII Oocytes
Garcia-Velasco, 2013 [48]	Retrospective, multicenter, observational study	38; N/A	Antagonist protocol	-
Raad, 2018 [49]	Retrospective Cohort	49; 70	Antagonist or long agonist protocols	7.2 ± 4.9
Cobo, 2020 [36]	Retrospective cohort study	485; 840	Antagonist or agonist protocols	5.5 ± 5.2
D’Argent, 2020 [50]	Prospective cohort study	108; 108	54 women were stimulated with an antagonist protocol; 54 with a PPOS protocol	-
Kim, 2020 [51]	Retrospective Cohort	34; 50	Antagonist protocol	4.1 ± 3.1
Santulli, 2021 [52]	Retrospective Cohort	146; 258	Long agonist, short agonist or antagonist protocol	6.0 ± 4.7
Legrand, 2021 [53]	Retrospective cohort	70; 113	Agonist or antagonist protocol	-
Yeon Hee Hong, 2021 [54]	Retrospective Cohort	62; 95	Antagonist protocol	3.0 (1.5–5.0)
Elizur, 2023 [55]	Retrospective Cohort	71; 138	Antagonist protocol	6 (3–10)
